# Haemoglobin A1c Time‐In‐Range and Mortality in Adults With Diabetes

**DOI:** 10.1002/edm2.70197

**Published:** 2026-03-29

**Authors:** Paul R. Conlin, Julia C. Prentice, David C. Mohr, Libin Zhang, Donglin Li, Erin Pleasants, Richard E. Nelson, Suma Vupputuri, Gregory A. Nichols

**Affiliations:** ^1^ VA Boston Healthcare System Boston Massachusetts USA; ^2^ Harvard Medical School Boston Massachusetts USA; ^3^ Boston University School of Medicine Boston Massachusetts USA; ^4^ National Center for Organizational Development, Veterans Health Administration Mason Ohio USA; ^5^ Boston University School of Public Health Boston Massachusetts USA; ^6^ VA Salt Lake City Healthcare System Salt Lake City Utah USA; ^7^ University of Utah Salt Lake City Utah USA; ^8^ Mid‐Atlantic Permanente Research Institute Rockville Maryland USA; ^9^ Kaiser Permanente Center for Health Research Portland Oregon USA

**Keywords:** haemoglobin A1c, haemoglobin A1c variability, mortality

## Abstract

**Introduction:**

Glycemic variability over time is a risk factor for diabetes complications. We studied a new measure of A1c stability, A1c time in range and its association with mortality in adults with diabetes.

**Methods:**

We conducted a retrospective cohort study of people with type 1 and type 2 diabetes from January 1, 2004 to December 31, 2018. Participants were 18 years or older with at least four A1c tests during a three‐year baseline. A1c time in range was calculated as the percentage of time during baseline when A1c levels were within person‐specific target ranges. We also calculated the percentage of time A1c levels were below target ranges to assess the effects of potential overtreatment. Adjusted models and instrumental variable models were used to measure associations between A1c time in range and mortality.

**Results:**

We studied 84,229 participants with mean age 58.3 years (SD 12.1). In adjusted Cox models, each 20% decrement in A1c time in range was associated with increased mortality (A1c time in range [0% < 20%] HR, 1.30; 95% CI 1.23–1.37). Instrumental variable models to control for unmeasured confounding also showed associations between reduced A1c time in range (0% < 20%) and mortality (HR, 1.28, 95% CI 1.22–1.35). Mortality risks were similar in subgroups with or without prevalent cardiovascular disease and insulin users but were greater in older adults (≥ 60 years). Greater (≥ 60%) A1c time below range was associated with increased mortality (HR, 1.35, 95% CI 1.29–1.41).

**Conclusions:**

Reduced A1c time in range and particularly greater time below range are associated with increased mortality in adults with diabetes. A1c stability within person‐specific ranges may be a risk factor for major outcomes in adults with diabetes.

## Graphical abstract

Reduced A1c time in range and particularly greater time below range are associated with increased mortality in adults with diabetes. A1c stability within person‐specific ranges may be a risk predictor for major outcomes in adults with diabetes.

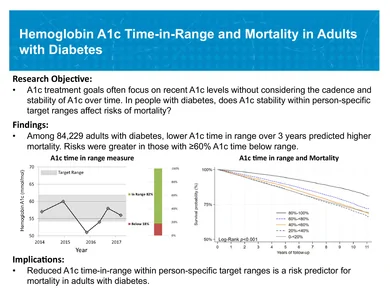

## 1 Introduction

Visit‐to‐visit glycemic variability is a risk factor for mortality and diabetes complications. Several observational studies showed that increased haemoglobin A1c (A1c) variability and fasting glucose variability are associated with mortality and diabetes complications [1, 2, 3, 4]. Incorporating these findings into clinical practice has been limited as there is no consensus about how to express longer‐term glycemic variability and causal links to adverse outcomes have not been confirmed.

Most diabetes clinical practice guidelines recommend maintaining A1c levels below certain thresholds based on health status, life expectancy and the presence or absence of comorbidities [5, 6, 7, 8]. Thus, clinicians often assume that merely reducing A1c levels over time is sufficient to lower risks of adverse outcomes, with less attention paid to the optimal A1c target range. Several observational studies showed a non‐linear, U‐shaped relationship between average A1c and mortality in persons with type 2 diabetes [9, 10]. This suggests that treatment strategies focusing only on reducing A1c levels without considering the risks and benefits from lower A1c levels or person‐specific A1c stability may lead to potential over‐treatment [11].

We developed a clinical measure of A1c stability within person‐specific target ranges, A1c time in range (A1c TIR), which is the percentage of time over 3 years that A1c levels are within target ranges based on age and comorbidities [12]. We showed that higher A1c TIR is associated with lower risks of mortality, cardiovascular disease and microvascular complications in older (≥ 65 years) military veterans with diabetes [12, 13, 14]. To broaden the generalizability of A1c TIR as a clinical measure, we sought to confirm these findings in other health systems and settings. This cohort study examines A1c TIR as a risk predictor for mortality in a large privately insured cohort of adults with diabetes and uses methods to limit the effects of confounding and bias.

## 2 Materials and Methods

### 2.1 Study Population

This retrospective cohort study was reviewed and approved by the Institutional Review Boards at Kaiser Permanente (KP) Northwest and the Department of Veterans Affairs (VA) Boston Healthcare System and was considered exempt from informed consent. Study participants were 18 years or older to be inclusive of all adults diagnosed with diabetes and drawn from KP Northwest and KP MidAtlantic States. Kaiser Permanente is an integrated healthcare system across the US that closely resembles the demographics of the US population, including the prevalence of diabetes [15]. We used electronic health records and associated administrative data from January 1, 2004 through December 31, 2018 to track participant enrollment over time. A diabetes diagnosis was defined by having at least two outpatient or one inpatient diagnosis code (ICD‐9‐CM: 250.XX, 357.2, 362.0X, 366.41 and ICD‐10‐CM: E10.X, E11.X, E13.X, where X indicates all sub‐codes) or a prescription for diabetes medications [16], with eligibility based on the date of a qualifying diagnosis or prescription. Participants were also required to have four or more A1c tests with none more than 12 months apart during a three‐year baseline period that started from January 1, 2005 through December 31, 2014, with follow‐up through December 31, 2018 Figure A1. All haemoglobin A1c assays were standardized to the National Glycohemoglobin Standardization Program. Enrollment occurred as soon as the person had a confirmed diabetes diagnosis and met the requirements for A1c test number and duration. Outcome assessment began immediately after the baseline.

### 2.2 Person‐Level A1c Time‐In‐Range (A1c TIR)

**FIGURE A2:**
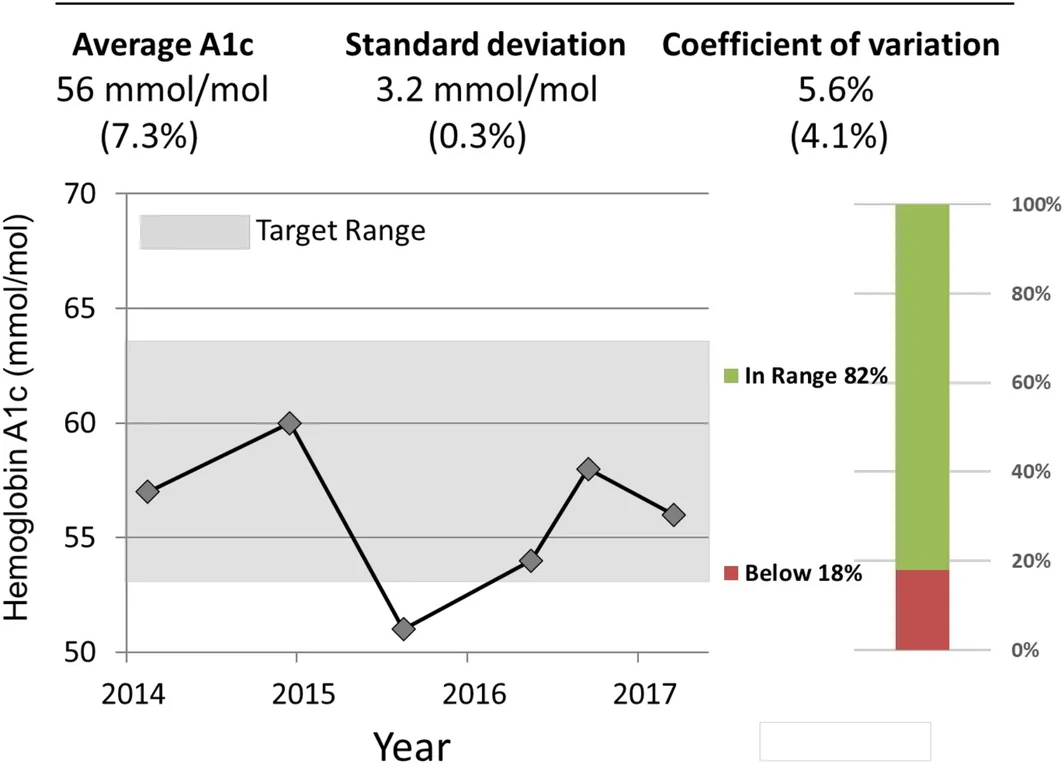
Calculating A1c time in range.

Person‐level A1c target ranges used the VA/Department of Defence Diabetes Clinical Practice Guideline [6]. Based on a person's predicted life expectancy and the presence and severity of comorbidities and diabetes complications, four A1c target ranges were applied: 42–53 mmol/mol (6.0%–7.0%), 53–64 mmol/mol (7.0%–8.0%), 58–69 mmol/mol (7.5%–8.5%) and 64–75 mmol/mol (8.0%–9.0%). We used clinical and administrative data to estimate life expectancy [17] and participants were grouped by less than 5, 5–10 and 10 or more years. The Diabetes Complications Severity Index (DSCI) [18] was used to identify diabetes complications and their severity from diagnosis codes and laboratory data. Life expectancy and prevalent diabetes complications were measured preceding each year of the 3 year baseline to set the person's A1c target range. Target ranges were updated yearly during baseline to adjust for changes in comorbidities or new diagnoses. A1c TIR was calculated by linear interpolation between A1c levels and test dates and expressed as the percentage of days A1c levels were within a person's individualized target range (Figure A2).

A1c TIR was expressed in five categories of decreasing time in range: 80%–100% (reference group), 60%–< 80%, 40%–< 60%, 20%–< 40% and 0%–< 20%. We also studied out of range levels by grouping participants into categories based on the percentage of days that A1c was within, below, or above their target range. A ≥ 60% threshold was used for each because this constituted a majority of time spent in the respective category. The groups were: ≥ 60% A1c TIR (reference group), ≥ 60% A1c time below range and ≥ 60% A1c time above range.

### 2.3 Covariates

Covariates included person‐level factors such as age, sex (self‐reported), race/ethnicity (self‐reported), DCSI score, comorbidities, medications and laboratory tests during baseline. The Elixhauser comorbidity index [19] measured comorbidities during baseline. Type 1 and type 2 diabetes were determined based upon diagnosis codes. Diabetes medications during baseline were categorized by class. Insulin use included basal and/or prandial insulin. Medication adherence was calculated as the medication possession ratio (i.e., days' supply/total number of days) for all prescribed diabetes medications, separated into those with or without ≥ 80% days of on‐hand medication supply.

Baseline laboratory measures included the participant's average A1c (expressed as mmol/mol and %), A1c standard deviation, number of A1c tests, serum creatinine, serum albumin, urine albumin‐to‐creatinine ratio and blood lipids (i.e., HDL, LDL, triglycerides). Baseline clinical measures included average blood pressure and body mass index, which were grouped using clinical criteria (e.g., low, normal, high or not reported).

Clinician characteristics (i.e., physician, nurse practitioner, physician assistant, other) were identified for those who ordered the most A1c tests for a given person, and if they were a primary care clinician. We controlled for the KP health system where the participant received care. Time trends were modelled based on the calendar quarter in which a person entered the outcome period.

### 2.4 Instrumental Variable

We used an instrumental variable to overcome confounding by unmeasured factors in the association of A1c TIR and outcomes. An instrumental variable is related to the treatment received but not related to the outcome except through its effect on receipt of treatment [20, 21]. Practice pattern variation is often used as an instrument [22, 23, 24]. We calculated clinician‐level A1c TIR as this directly affects patient‐level A1c TIR. We computed all patient‐level A1c TIR for each clinician's patients during baseline and averaged the values. If a clinician had fewer than 10 patients, we used the A1c TIR for all clinicians at the individual treatment site.

We also used a falsification test to validate the assumption that clinician A1c TIR influences diabetes‐related outcomes through its effects on person‐level A1c TIR [25]. We analysed the effects of clinician A1c TIR in people without diabetes who were diagnosed with asthma, chronic obstructive pulmonary disease (COPD) and heart failure [26]. We predicted mortality in this group using clinician A1c TIR and all covariates.

### 2.5 Outcomes

All‐cause mortality was the primary outcome and was determined using data from the Social Security Administration, state‐level vital health status records and electronic health record information. During follow‐up, individuals were censored if they were disenrolled from the KP healthcare system or at the end of the study period.

### 2.6 Statistical Analysis

Person‐level characteristics and clinical variables were described using means and proportions. Adjusted Cox proportional hazards regression models were used to estimate the effects of A1c TIR on time to mortality. We assessed the Cox models for violation of the proportional hazards assumption with Schoenfeld residuals and estimated the effects of A1c TIR on mortality with accelerated failure time models. Instrumental variable models used a 2‐stage residual inclusion approach to analyse associations between A1c TIR and mortality. The first stage applied linear regression of person‐level A1c TIR, the instrumental variable (i.e., clinician‐level A1c TIR), and all covariates. F‐statistics from the first stage were used as an indicator of the strength of the instrument, with values less than 10 generally indicative of a weak instrument [24]. Clinician A1c TIR was positively associated with person‐level A1c TIR in first‐stage models of mortality (*F* = 472, *p* < 0.001). The second stage used a multivariable Cox proportional hazards regression including residuals from the first stage as an additional covariate.

We conducted several additional analyses. We examined a higher threshold (≥ 80%) for the association of A1c time below and above range and mortality. We also studied A1c TIR as a risk predictor in higher‐risk subgroups such as people with prevalent cardiovascular disease, older adults and insulin or sulfonylurea users. All adjusted models included the main explanatory variables and all covariates. Analyses were conducted using STATA 15.

## 3 Results

The study cohort included 84,229 people with diabetes (Table 1). The mean age was 58.3 (SD 12.1) years. About one‐half were female and non‐White race/ethnicity. Average A1c was 57 mmol/mol (SD 14) (7.4%, SD 1.3%). The number (percentage) of participants assigned to each of four A1c target ranges at the end of baseline were: 53,780 (63.9%), target range 42–53 mmol/mol (6.0%–7.0%); 15,336 (18.2%), target range‐ 53–64 mmol/mol (7.0%–8.0%); 10.947 (13.0%), target range‐ 58–69 mmol/mol (7.5%–8.5%); and 4136 (4.9%), target range 64–75 mmol/mol (8.0%–9.0%) (Table A1). About 39% (33,137) of participants had ≥ 60% A1c TIR. During an average follow‐up of 6.8 years (SD 3.4), 16,003 participants (19%) died.

**TABLE 1:** Baseline characteristics of the study population (*N* = 84,229).

Characteristic	Mean (SD) or *n* (%)
Age at end of baseline, mean (SD) y	58.3 (12.1)
Sex: Female	42,015 (49.8%)
Diabetes: type 1	1731 (2.1%)
Race/Ethnicity	
White	44,799 (53.2%)
Black	23,058 (27.4%)
Hispanic	5442 (6.5%)
Asian	5333 (6.3%)
Other	5597 (6.6%)
Clinical measures	
HbA1c, mean (SD)	57 mmol/mol (14) 7.4% (1.3)
HbA1c SD, mean (SD)	8 mmol/mol (7) 0.73% (0.63)
Diabetes Complications Severity Index score, mean (SD)	2.0 (2.1)
Body Mass Index, mean (SD)	33.4 (7.6)
Medications	
Sulfonylurea	40,290 (47.8%)
Metformin	56,950 (67.6%)
Insulin	22,124 (26.3%)
Thiazolidinedione	8276 (9.8%)
Other	2147 (2.6%)
Diabetes medication adherence – Proportion of days covered ≥ 80%	58,635 (69.6%)
Comorbidities	
Cardiovascular disease	23,946 (28.4%)
Cerebrovascular disease	7536 (9.0%)
Heart failure	8878 (10.5%)
A1c time in range	
80%–100%	21,057 (25.0%)
60% < 80%	12,080 (14.3%)
40% < 60%	11,032 (13.1%)
20% < 40%	12,553 (14.9%)
0% < 20%	27,507 (32.7%)

Abbreviations: HbA1c, haemoglobin A1c; SD, standard deviation.

### 3.1 A1c Time‐In‐Range and Clinical Outcomes

**FIGURE 1:**
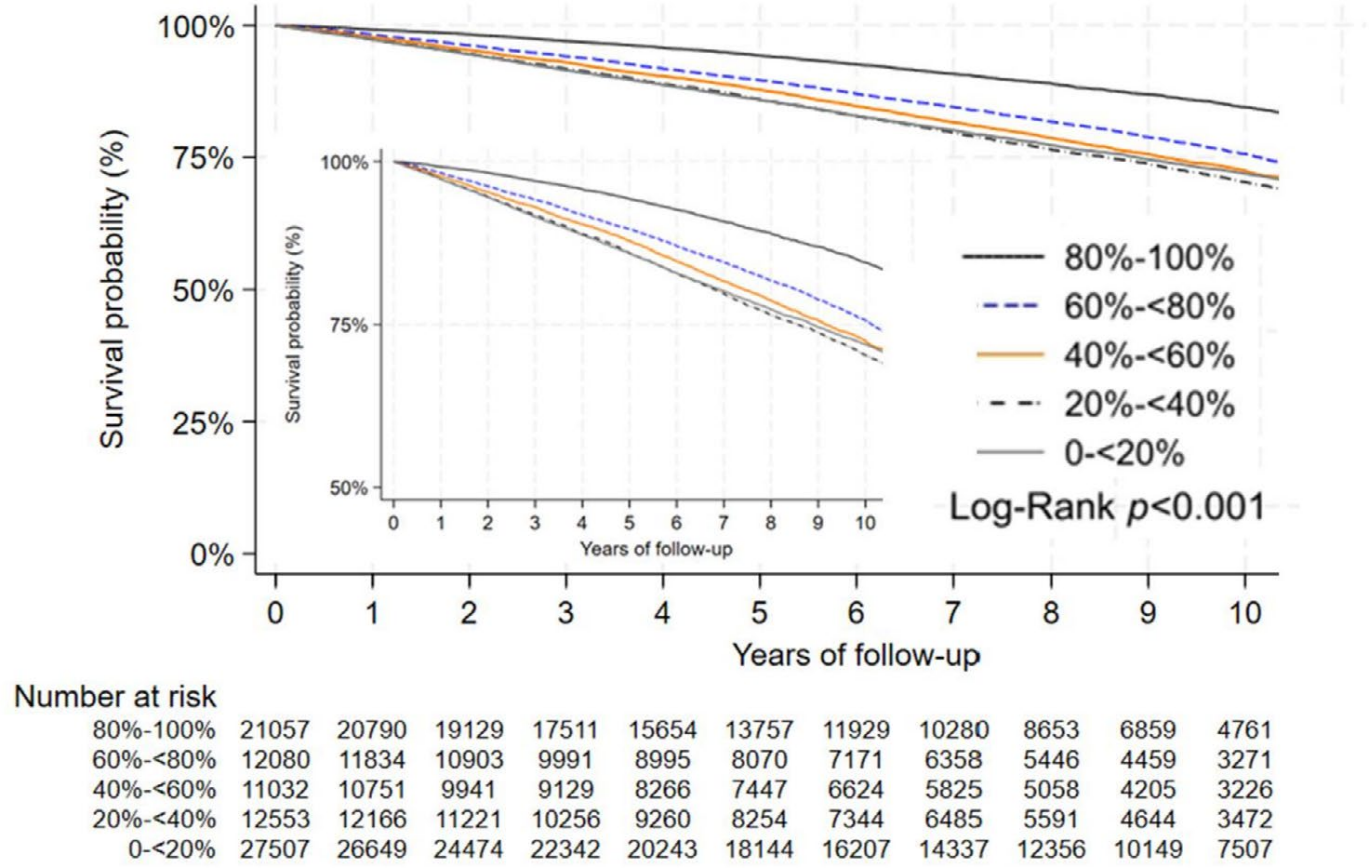
Kaplan‐Meir estimates for time to mortality by A1c Time‐in‐Range. Kaplan‐Meir estimates for time to mortality by 20% decrements in A1c time‐in‐range. The insert displays the curves using the upper half of the survival probability axis.

When compared to higher A1c TIR (80%–100%), decrements in A1c TIR were associated with greater risk of mortality. Results from unadjusted Cox proportional hazards models showed a doubling of mortality risk with progressively lower A1c TIR (Table 2). Adjusted Cox models showed that low (0 < 20%) A1c TIR had the greatest mortality risks (HR 1.30, 95% CI 1.23–1.37) (Table 2 and Table A2). Tests of the proportional hazards assumption showed that the relative risk of mortality was not constant over time. Therefore, we estimated mortality using accelerated failure time models, which showed similar results (Table A3). Instrumental variable models also showed that reduced A1c TIR (0 < 20%) was associated with increased risk of mortality (HR 1.28, 95% CI 1.22–1.35) (Table 2). Survival probability curves demonstrated increasing mortality risks with lower A1c TIR (Figure 1).

**TABLE 2:** Adjusted hazard ratios (95% CI) and instrumental variable models predicting mortality by A1c time in range.

A1c Time in Range	Mortality		
	Unadjusted Cox model	Adjusted Cox model	IV model
80%–100% (*N* = 21,057)	1.0	1.0	1.0
60 < 80% (*N* = 12,080)	1.72 (1.62, 1.82)	1.18 (1.11, 1.35)	1.16 (1.09, 1.23)
40% < 60% (*N* = 11,032)	2.00 (1.88, 2.12)	1.23 (1.15, 1.30)	1.20 (1.13, 1.28)
20% < 40% (*N* = 12,553)	2.21 (2.09, 2.33)	1.22 (1.15, 1.30)	1.20 (1.13, 1.28)
0% < 20% (*N* = 27,507)	2.10 (2.00, 2.20)	1.30 (1.23, 1.73)	1.28 (1.22, 1.35)

Abbreviations: A1c, haemoglobin A1c; IV, instrumental variable.

### 3.2 Instrumental Variable and Clinical Outcomes in People Without Diabetes

To assess the specificity of the instrumental variable in predicting diabetes outcomes, we assessed the effects of clinician‐level A1c TIR on mortality in a group of people without diabetes. We identified 84,782 participants with COPD, asthma and heart failure and without diabetes (Table A4). Clinician A1c TIR did not predict mortality in people without diabetes (Table A5).

### 3.3 A1c Time‐In‐Range and Clinical Outcomes in Subgroups

People with cardiovascular disease, older adults and those using insulin or sulfonylureas may have different risk profiles related to A1c TIR. Therefore, we conducted stratified analyses in each group. Lower A1c TIR predicted mortality in people using insulin, insulin or sulfonylureas, or oral diabetes medications (without sulfonylureas) (Table A6) as well as those with or without prevalent cardiovascular disease (Table A7). The association of A1c TIR with mortality was most evident in older adults (≥ 60 years) (Tables A8 and A9) and people with type 2 diabetes vs. type 1 diabetes (Table A10).

The adjusted Cox models controlled for A1c standard deviation (SD) as a measure of A1c variability, but we also assessed the independent effect of A1c SD in predicting mortality. Increased A1c SD was associated with mortality when assessed in adjusted models and when stratified by quartiles (Tables A11 and A12).

### 3.4 A1c Time Out of Range and Mortality

To assess the effects of greater A1c time out of range, we grouped participants into distinct categories based on having most of the time (≥ 60%) with A1c in range, below range, or above range during baseline. The number (percentages) of people in each category were: 31,137 (39%) with ≥ 60% A1c TIR, 11,975 (14%) with ≥ 60% A1c time below range and 25,810 (30.6%) with ≥ 60% A1c time above range (Table A13). Greater (≥ 60%) A1c time below range as a marker of potential overtreatment was associated with increased risk of mortality (HR 1.35, 95% CI 1.29–1.41) (Table 3). We also assessed mortality using a higher A1c time below range threshold (≥ 80%) and risk was higher (HR 1.53, 95% CI 1.44–1.62). Greater time above range had somewhat higher mortality risk above the 80% threshold.

**TABLE 3:** Adjusted hazard ratios (95% CI) predicting mortality by ≥ 60% and ≥ 80% time below and time above range.

Category	Mortality
≥ 60% A1c Time in Range (Reference) (*n* = 33,137)	1.0
≥ 60% A1c Time Below Range (*n* = 11,975)	1.35 (1.29, 1.41)
≥ 60% A1c Time Above Range (*n* = 25,810)	0.97 (0.92, 1.02)
≥ 80% A1c Time in Range (Reference) (*n* = 21,057)	1.0
≥ 80% A1c Time Below Range (*n* = 7644)	1.53 (1.44, 1.62)
≥ 80% A1c Time Above Range (*n* = 7644)	1.06 (0.99, 1.14)

We conducted stratified analyses within each A1c target range category. The group with ≥ 60% A1c time below range had higher mortality risks for A1c target ranges greater than 53 mmol/mol (7.0%), although there were fewer deaths in the target range group 42–53 mmol/mol (6.0%–7.0%) (Table A14).

## 4 Discussion

We showed that lower A1c TIR over time is associated with higher mortality risk in adults with diabetes with a small dose effect for each 20% decrement in A1c TIR. When individuals with low A1c TIR were separated into those with mostly in range, below range and above range levels, ≥ 60% A1c time below range was associated with increased risks of mortality. These findings suggest that A1c stability within person‐specific target ranges is associated with lower risk, and increased time below range is a risk predictor for mortality in adults with diabetes.

Various measures have been proposed to express A1c variability over time such as absolute A1c changes (e.g., 5 mmol/mol [0.5%]), standard deviation, or coefficient of variation [1, 2, 3, 4, 26]. Increasing levels of each parameter are associated with risks of adverse outcomes including mortality. However, these measures have some limits by not accounting for (1) time of exposure to A1c variability, (2) conveying the direction of A1c deviations, or (3) that higher or lower A1c target ranges may be appropriate for a person's unique clinical characteristics. A1c TIR captures these parameters. We also acknowledge that time in range and out of range metrics are applied to continuous glucose monitoring to track intra‐day and day‐to‐day glucose readings using fixed ranges. These metrics may be complimentary with A1c TIR. Use of continuous glucose monitors is growing, but data are lacking on the effects of short‐term glycemic variability on major adverse outcomes. Most people with type 2 diabetes continue to have glycemic management measured with A1c levels. Thus, A1c TIR may be useful to track and evaluate the effectiveness of glycemic treatment strategies over time and the risks of major adverse outcomes, particularly when people have low A1c TIR and increased time below range.

Several large clinical trials showed that targeting lower A1c levels over time in people with established type 2 diabetes is associated with lower risks of microvascular complications [27, 28] and treating people with new‐onset diabetes to lower A1c levels may accrue mortality benefits over time [29]. However, near‐normal A1c levels may not be appropriate for all individuals based on their age and comorbidities. Most diabetes guidelines recommend adjusting glycemic treatment goals based on potential risks and benefits [5, 6, 7, 8]. Higher A1c levels are acceptable when individuals have comorbidities and/or shorter life expectancy. Our findings support this individualization as well as the use of A1c target ranges with upper and lower bounds. Study participants with reduced A1c TIR had increased risk of mortality, and risks were particularly increased when the majority of time was spent with A1c levels below range. Mortality risks were increased in the subgroups of people treated with non‐sulfonylurea oral medications, insulin, or any combination of insulin or sulfonylurea medications. These findings do not suggest that treatment‐related hypoglycemia is a mediator of the risks associated with reduced A1c TIR. Nonetheless, these results support a role for tailoring diabetes treatments and goals and considering the risks of potential overtreatment in adults with greater comorbidity burdens.

Our study used adjusted Cox models and instrumental variable models to reduce the effects of unmeasured confounding in the results. Unmeasured variables may include self‐care behaviours (e.g., exercise and nutrition), psychosocial factors and clinicians' treatment practices. Instrumental variable models estimate the effects in the subset of participants whose A1c TIR is influenced by their clinician's A1c TIR, which is termed the local average treatment effect [20]. Multivariable models estimate an average treatment effect across all participants, whether or not their A1c TIR is influenced by the clinician's A1c TIR. Our use of these different models was complementary, and mortality risks were similar across all models.

Our study has several strengths. We studied a cohort of adults with diabetes from a large private healthcare system. The study design used a baseline period to capture prevalent comorbidities and calculate A1c TIR before assessing outcome measures to minimize reverse causation. We used adjusted models that controlled for many demographic and clinical characteristics including age, comorbidities, medications, average A1c, A1c standard deviation and number of A1c tests. The instrumental variable models added quasi‐experimental methods to further limit potential confounding. We also used a non‐diabetes falsification sample to test the specificity of the instrumental variable. The long duration of follow‐up allowed us to identify many mortality outcomes. Nonetheless, our study has several limitations. The study period spanned 15 years and clinicians may have targeted different A1c levels or used different diabetes medications over time based on prevailing clinical guidelines. Although fewer participants were receiving diabetes medications associated with lower risk of hypoglycemia, mortality risks were similar among insulin and non‐insulin users. Study eligibility criteria required four or more A1c tests during baseline to calculate A1c TIR, and this may have identified patients with more interactions with the healthcare system, leading to selection or surveillance bias. Increased A1c TAR was not associated with increased mortality risk, although this is likely due to the younger age of this study population. We previously showed that ≥ 60% A1c TAR is a risk factor for mortality and cardiovascular outcomes in older adults [30]. The study population was drawn from a healthcare system in the United States and results may not translate to other health systems in other countries. The study had a small sample of people with type 1 diabetes and results were non‐significant in this subgroup. Such subgroup analyses may be limited by study power so these findings should be considered inconclusive. Lastly, this is still an observational study, and some unmeasured factors cannot be controlled despite the use of statistical methods to adjust for confounding. Therefore, we cannot claim causality between A1c TIR and the observed outcomes or assert that prospectively increasing A1c TIR will reduce these outcomes. Fully addressing this causal question requires a randomized trial, but observational studies may help define treatment estimates.

In conclusion, reduced A1c TIR is associated with higher risks of mortality. These results suggest a role for A1c stability within individualized target ranges as a risk factor for major adverse outcomes in adults with diabetes.

## Author Contributions

All authors made contributions to the intellectual content of the paper, including the design of the study (P.R.C., J.C.P., D.C.M., R.E.N., S.V., G.A.N.), the acquisition of data (L.Z., D.L., S.V., G.A.N.), the statistical analyses (P.R.C., J.C.P., D.C.M., L.Z., R.E.N.), interpretation of the data (all authors), and the drafting and critical revision of the manuscript (all authors). P.R.C. is the guarantor of this work and, as such, had full access to all the data in the study and takes responsibility for the integrity of the data and the accuracy of the data analysis.

## Funding

Supported by the National Institutes of Health, National Institute of Diabetes and Digestive and Kidney Diseases (R01 DK114098) and Department of Veterans Affairs, Office of Research and Development, Health Systems Research (IIR 15‐116 and I01 HX003527). Data were obtained with support from VA Information Resource Center (VIReC), VA/CMS Data for Research Projects SDR 02‐23 and 98‐004.

## Conflicts of Interest

The authors declare no conflicts of interest.

## Figures and Tables

**FIGURE A1:**
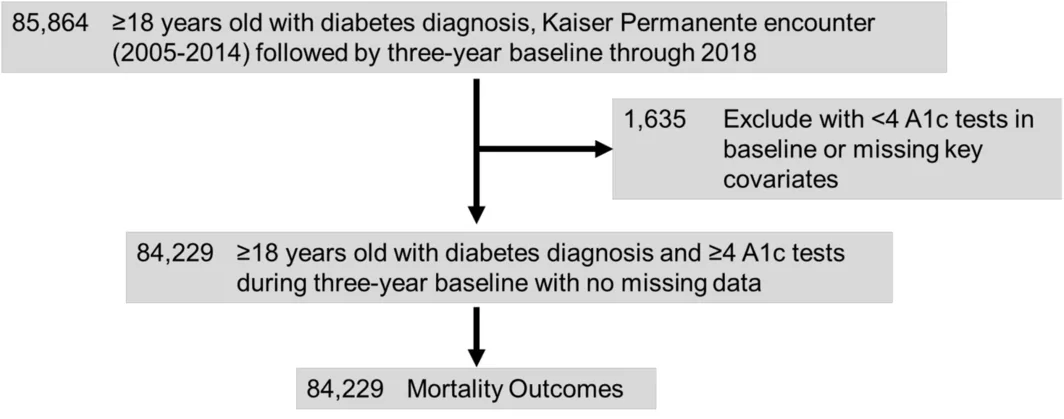
Flow chart of participants. A1c, haemoglobin A1c.

## Data Availability

The data that support the findings of this study are only available from the corresponding author with the permission of the US Department of Veterans Affairs, Kaiser Permanente, and Medicare. The data are not publicly available due to privacy or ethical restrictions.

## Appendix A Table of Contents

**Table:**

Item	Content	Page
Figure A1	Flow Chart of Participants	2
Figure A2	Calculating A1c time in range	3
Table A1	Distribution of Participants by A1c Target Ranges	4
Table A2	Full Model with Adjusted Hazard Ratios (95% CI) Predicting Mortality by A1c Time in Range	5
Table A3	Adjusted Accelerated Failure Time Model (95% CI) Predicting Mortality by A1c Time in Range	9
Table A4	Baseline Characteristics of Patients without Diabetes Falsification Sample	10
Table A5	Instrumental Variable (95% CI) Predicting Mortality in Patients without Diabetes	11
Table A6	Adjusted Hazard Ratios (95% CI) Predicting Mortality by A1c Time in Range Stratified by Oral Diabetes Medications and Insulin Use	12
Table A7	Adjusted Hazard Ratios (95% CI) Predicting Mortality by A1c Time in Range Stratified by Cardiovascular Disease	13
Table A8	Adjusted Hazard Ratios (95% CI) Predicting Mortality by A1c Time in Range including Age	14
Table A9	Adjusted Hazard Ratios (95% CI) Predicting Mortality by A1c Time in Range Stratified by Age	15
Table A10	Adjusted Hazard Ratios (95% CI) Predicting Mortality by A1c Time in Range Stratified by Diabetes Type	16
Table A11	Adjusted Hazard Ratios (95% CI) Predicting Mortality for A1c Time in Range Stratified by A1c Standard Deviation	17
Table A12	Adjusted Hazard Ratios (95% CI) Predicting Mortality by Quartiles of A1c SD	18
Table A13	Baseline Characteristics of the Study Population by ≥ 60% A1c Time in Range, ≥ 60% A1c Time Below Range, and ≥ 60% A1c Time Above Range	19
Table A14	Adjusted Hazard Ratios (95% CI) Predicting Mortality by ≥ 60% A1c Time in Range and ≥ 60% A1c Time below Range Stratified by A1c Target Ranges	21

This example shows a 70 year‐old person who entered the study sample on 2/1/2014. A one‐year pre‐baseline period was used to collect diagnosis codes, lab values and utilization data. Based on predicted life expectancy, comorbidities and complications, this person was predicted to have 5–10 years of life expectancy and had a DCSI score of 1.0. These correspond with an A1c target range of 53–64 mmol/mol (7.0%–8.0%) (see Table A1). They developed no new comorbidities or complications to affect life expectancy or DCSI score over the ensuing 3 years, so their target range remained unchanged. They had 6 A1c tests during the baseline period. Linear interpolation between A1c values was applied and the number of days A1c levels were in the target range was calculated. A1c levels were in range for 898/1095 days (82%), below range for 197/1095 days (18%), and there was no time above range. For illustration purposes, we also show A1c average, standard deviation and coefficient of variation.

**TABLE A1:** Distribution of participants by A1c target ranges (*N* = 84,229).

	Diabetes complications		
	Absent or Mild (DCSI = 0–1)	Moderate (DCSI = 2–3)	Advanced (DCSI ≥ 4)
Life expectancy	A1c Target Range		
> 10 years	42–53 mmol/mol (6.0%–7.0%) *n* = 53,780 (63.9%)	53–64 mmol/mol (7.0%–8.0%) *n* = 11,871 (14.1%)	58–69 mmol/mol (7.5%–8.5%) *n* = 2400 (2.9%)
5 to 10 years	53–64 mmol/mol (7.0%–8.0%) *n* = 3495 (4.2%)	58–69 mmol/mol (7.5%–8.5%) *n* = 4730 (5.6%)	58–69 mmol/mol (7.5%–8.5%) *n* = 3817 (4.5%)
< 5 years	64–75 mmol/mol (8.0%–9.0%) *n* = 527 (0.6%)	64–75 mmol/mol (8.0%–9.0%) *n* = 1156 (1.4%)	64–75 mmol/mol (8.0%–9.0%) *n* = 2453 (2.9%)

*Note:* Life expectancy was based on predicting the likelihood of mortality in < 5 years, 5–10 years and ≥ 10 years during baseline.

Abbreviations: A1c, haemoglobin A1c; DCSI, Diabetes Complication and Severity Index.

**TABLE A2:** Full model with adjusted hazard ratios (95% CI) predicting mortality by A1c time in range (*N* = 84,229).

Parameters	Hazard ratio	95% confidence interval	*p*
Patient A1c Time in Range (Reference: 80%–100% Time In Range)			
60% < 80%	1.18	(1.11–1.04)	< 0.001
40% < 60%	1.23	(1.08–1.12)	< 0.001
20% < 40%	1.22	(1.04–1.07)	< 0.001
0% < 20%	1.30	(1.23–1.37)	< 0.001
Sex (reference: Female)	0.90	(0.87–0.94)	< 0.001
Race/Ethnicity (reference: White)			
Black	0.79	(0.75–0.83)	< 0.001
Hispanic	0.66	(0.60–0.73)	< 0.001
Asian	0.54	(0.49–0.60)	< 0.001
Other	1.86	(1.76–1.96)	< 0.001
Age at Follow‐up (reference: < 60 years)			
> 60 years	3.1	(3.00–3.22)	< 0.001
Diabetes Type reference: type 2 diabetes			
Type 1 diabetes	0.75	(0.67–0.84)	< 001
Provider type (reference: physician)			
Physician Assistant/Nurse Practitioner	0.87	(0.77–0.98)	0.02
Other	0.96	(0.96–1.01)	0.44
Number of A1c tests during the Baseline	0.99	(0.98–0.99)	0.002
Standard Deviation of All A1c tests during the baseline	1.04	(1.00–1.08)	0.02
Diabetes complication severity index score	1.13	(1.12–1.14)	< 0.001
Albumin/Creatinine Ratio (Urine) (reference: < 30 mg/g)			
30–300	1.30	(1.25–1.36)	< 0.001
> 300	1.54	(1.43–1.66)	< 0.001
Missing	1.30	(1.23–1.38)	< 0.001
LDL (reference: < 100 mg/dL)			
100–159	1.00	(0.97–1.04)	0.75
≥ 160	0.99	(0.90–1.10)	0.96
Missing	1.20	(0.83–1.71)	0.32
HDL (reference: < 40 mg/dL)			
40–60	0.95	(0.91–0.99)	0.008
≥ 60	0.93	(0.88–0.99)	0.014
Missing	0.95	(0.64–1.41)	0.81
Creatinine (reference: < 0.6 mg/dL)			
0.6–1.2	1.11	(0.95–1.30)	0.18
> 1.2	1.26	(1.07–1.49)	< 0.001
Missing	0.67	(0.28–1.68)	0.41
Albumin (reference: < 3.5 g/dL)			
≥ 3.5	0.75	(0.69–0.81)	< 0.001
Missing	0.70	(0.64–0.76)	< 0.001
BMI (reference: 18.5–24.9 kg/m [2])			
< 18.5	1.94	(1.56–2.43)	< 0.001
25–29.9	0.75	(0.71–0.79)	< 0.001
30–39.9	0.68	(0.64–0.71)	< 0.001
≥ 40	0.69	(0.64–0.74)	< 0.001
Missing	0.94	(0.88–1.01)	0.12
Triglycerides (reference: < 200 mg/dL)			
> = 200	0.6	(0.92–0.99)	0.037
Missing	1.62	(1.15–2.30)	0.005
Blood Pressure Systolic (reference: < 140 mmHg)			
140–179	1.19	(1.14–1.23)	< 0.001
≥ 180	2.01	(1.39–2.90)	< 0.001
Missing	1.31	(0.96–1.78)	0.10
Medications (reference: no)			
Alpha‐glucosidase Inhibitors	1.05	(0.91–1.20)	0.51
Metformin	0.78	(0.75–0.81)	< 0.001
Insulin	1.09	(1.04–1.13)	< 0.001
Multidrug	0.85	(0.75–0.98)	0.02
Sulfonylureas	1.07	(1.03–1.11)	< 0.001
Thiazolidinediones	0.95	(0.88–0.99)	0.048
Medication Adherence: Any Diabetes Medications with Proportion of Days Covered ≥ 80% (reference: < 80%)	1.07	(1.03–1.11)	0.001
Heart failure	1.46	(1.40–1.53)	< 0.001
Cardiac arrhythmias	1.15	(1.11–1.19)	< 0.001
Valvular disease	1.09	(1.05–1.14)	< 0.001
Pulmonary Hypertension	1.10	(1.06–1.14)	< 0.001
Peripheral vascular Disorders	1.10	(1.06–1.16)	< 0.001
Uncomplicated hypertension	1.27	(1.20–1.35)	< 0.001
Complicated hypertension	0.94	(0.97–1.01)	0.12
Paralysis	1.11	(1.00–1.21)	0.03
Other neurological disorders	1.21	(1.14–1.27)	< 0.001
Pulmonary circulation disorders	1.22	(1.15–1.29)	< 0.001
Hypothyroidism	1.06	(1.01–1.10)	0.001
Liver disease	1.15	(1.08–1.22)	< 0.001
Peptic ulcer disease	1.00	(0.92–1.10)	0.90
Human immunodeficiency virus	1.10	(0.86–1.40)	0.44
Lymphoma	1.43	(1.26–1.61)	< 0.001
Metastatic cancer	2.28	(2.10–2.50)	< 0.001
Tumour	1.32	(1.26–1.38)	< 0.001
Rheumatoid arthritis/collagen vascular disease	1.10	(1.03–1.17)	0.002
Coagulopathy	1.15	(1.08–1.23)	< 0.001
Obesity	0.94	(0.86–1.01)	0.12
Weight loss	1.07	(1.00–1.14)	0.02
Fluid and electrolyte disorders	1.17	(1.12–1.21)	< 0.001
Blood loss anaemia	1.02	(0.93–1.12)	0.68
Anaemia	1.06	(1.00–1.11)	0.02
Alcohol abuse	1.20	(1.11–1.29)	< 0.001
Drug abuse	1.07	(0.99–1.15)	0.08
Psychosis	1.27	(1.18–1.36)	< 0.001
Depression	1.01	(0.98–1.05)	0.52

Abbreviations: BMI, body mass index; LDL, low‐density lipoproteins; HDL, high‐density lipoproteins.

**TABLE A3:** Adjusted accelerated failure time model (95% CI) predicting mortality by A1c time in range.

A1c Time in Range	Mortality (*n* = 84,229)
80%–100% (Reference)	1.0
60% < 80%	1.18 (1.11, 1.25)
40% < 60%	1.22 (1.14, 1.30)
20% < 40%	1.22 (1.15, 1.29)
0% < 20%	1.30 (1.23, 1.37)

*Note:* Models included all covariates.

Abbreviation: A1c, haemoglobin A1c.

**TABLE A4:** Baseline characteristics of patients without diabetes–falsification sample.

Characteristics (*n* = 84,782)	Mean (SD) or *n* (%)
Age, mean (SD) y	72.3 (5.7)
Sex: Female	36,371 (42.9%)
Race/Ethnicity	
White	17,273 (74.5%)
Black	3099 (13.4%)
Hispanic	40 (0.2%)
Asian	608 (2.3%)
Other	2152 (9.3%)
Cardiovascular disease	12,913 (55.7%)
Cerebrovascular disease	3159 (13.6%)
Heart failure	1601 (7.5%)
BMI, mean (SD)	29.1 (6.2)

**TABLE A5:** Instrumental variable (95% CI) predicting mortality in patients without diabetes.

Clinician A1c time in range	Mortality
80%–100% (Reference)	1.0
60% < 80%	1.32 (0.65, 2.66)
40% < 60%	1.31 (0.65, 2.63)
20% < 40%	1.33 (0.66, 2.67)
0% < 20%	1.40 (0.69, 2.82)

Abbreviation: A1c, haemoglobin A1c.

**TABLE A6:** Adjusted hazard ratios (95% CI) predicting mortality by A1c time in range stratified by oral diabetes medications and insulin use.

A1c time in range	Mortality		
	Oral diabetes medications (without sulfonylureas) (*n* = 32,924)	Insulin users (*n* = 22,124)	Insulin or sulfonylurea users (*n* = 51,288)
80%–100% (Reference)	1.0	1.0	1.0
60% < 80%	1.11 (0.99, 1.23)	1.13 (1.01, 1.27)	1.08 (1.00, 1.17)
40% < 60%	1.25 (1.11, 1.41)	1.15 (1.00, 1.25)	1.08 (0.99, 1.17)
20% < 40%	1.14 (1.02, 1.27)	1.14 (1.00, 1.23)	1.11 (1.03, 1.21)
0% < 20%	1.19 (1.07, 1.31)	1.21 (1.00, 1.22)	1.10 (1.02, 1.18)

*Note:* Models included all covariates.

Abbreviation: A1c, haemoglobin A1c.

**TABLE A7:** Adjusted hazard ratios (95% CI) predicting mortality by A1c time in range stratified by cardiovascular disease.

A1c time in range	Mortality	
	No cardiovascular disease	Prior cardiovascular disease
80%–100% (Reference)	1.0	1.0
60% < 80%	1.15 (1.06, 1.25)	1.09 (1.00, 1.20)
40% < 60%	1.25 (1.14, 1.36)	1.13 (1.04, 1.25)
20% < 40%	1.14 (1.05, 1.24)	1.19 (1.09, 1.30)
0% < 20%	1.30 (1.20, 1.40)	1.26 (1.17, 1.37)

*Note:* Models included all covariates.

Abbreviation: A1c, haemoglobin A1c.

**TABLE A8:** Adjusted hazard ratios (95% CI) predicting mortality by A1c time in range including age.

A1c time in range	Mortality
80%–100% (Reference)	1.0
60% < 80%	1.15 (1.08, 1.22)
40% < 60%	1.23 (1.15, 1.31)
20% < 40%	1.23 (1.16, 1.30)
0% < 20%	1.41 (1.33, 1.48)
Age (mean)	
< 60 years (Reference)	1.0
≥ 60 years	3.20 (3.07, 3.33)

*Note:* Models included all covariates.

Abbreviation: A1c, haemoglobin A1c.

**TABLE A9:** Adjusted hazard ratios (95% CI) predicting mortality by A1c time in range stratified by age.

A1c time in range	Mortality	
	Age < 60 years	Age ≥ 60 years
80%–100% (reference)	1.0	1.0
60% < 80%	1.07 (0.94, 1.23)	1.24 (1.16, 1.32)
40% < 60%	1.03 (0.91, 1.18)	1.33 (1.24, 1.43)
20% < 40%	1.05 (0.92, 1.19)	1.33 (1.25, 1.42)
0% < 20%	1.02 (0.91, 1.15)	1.50 (1.41, 1.59)

*Note:* Models included all covariates.

Abbreviation: A1c, haemoglobin A1c.

**TABLE A10:** Adjusted hazard ratios (95% CI) predicting mortality by A1c time in range stratified by diabetes type.

Type 2 diabetes		Type 1 diabetes	
A1c time in range	Mortality	A1c time in Range	Mortality
80%–100% (Reference) (*n* = 20,798)	1.0	80–100% (Reference) (*n* = 259)	1.0
60% < 80%	1.18	60% < 80%	0.73
(*n* = 11,879)	(1.11, 1.26)	(*n* = 201)	(0.46, 1.15)
40% < 60%	1.24	40% < 60%	0.76
(*n* = 10,809)	(1.16, 1.31)	(*n* = 223)	(0.49, 1.18)
20% < 40%	1.24	20% < 40%	0.85
(*n* = 12,305)	(1.16, 1.31)	(*n* = 248)	(0.85, 1.33)
0% < 20%	1.31	0% < 20%	0.90
(*n* = 26,707)	(1.25, 1.39)	(*n* = 800)	(0.62, 1.31)

**TABLE A11:** Adjusted hazard ratios (95% CI) predicting mortality for A1c time in range stratified by A1c standard deviation.

A1c time in range	Mortality
80%–100% (Reference)	1.0
60% < 80%	1.12 (1.05, 1.19)
40% < 60%	1.19 (1.12, 1.27)
20% < 40%	1.20 (1.13, 1.27)
0% < 20%	1.33 (1.27, 1.41)
A1c SD (mean)	
Below mean (Reference)	1.0
Above mean	1.13 (1.09, 1.18)

*Note:* Models included all covariates.

Abbreviations: A1c, haemoglobin A1c; SD, standard deviation.

**TABLE A12:** Adjusted hazard ratios (95% CI) predicting mortality by quartiles of A1c SD.

A1c SD Quartiles	Mortality *n* = 84,229
A1c SD Quartile 1 (Reference)	1.0
A1c SD Quartile 2	1.02 (0.98, 1.08)
A1c SD Quartile 3	1.05 (1.00, 1.11)
A1c SD Quartile 4	1.12 (1.05, 1.19)

*Note:* Models included all covariates.

Abbreviations: A1c, haemoglobin A1c; SD, standard deviation.

**TABLE A13:** Baseline characteristics of the study population by ≥ 60% A1c time in range, ≥ 60% A1c time below range, and ≥ 60% A1c time above range.

Characteristic	All participants (*n* = 84,229)	≥ 60% A1c Time in range (*n* = 33,137)	≥ 60% A1c Time below range (*n* = 11,975)	≥ 60% A1c Time above range (*n* = 25,810)
	Mean (SD) or *N* (%)			
Age at end of baseline (years)	58.3 (12.1)	58.9 (10.8)	67.9 (12.3)	52.9 (10.7)
Sex: Men	42,015 (49.8%)	17,585 (53.1%)	5616 (46.8%)	13,183 (51.1%)
Type 1 diabetes	1731 (2.1%)	469 (1.4%)	152 (1.3%)	851 (1.3%)
Race/Ethnicity				
White	44,799 (53.2%)	17,816 (53.7%)	8058 (62.3%)	11,517 (44.6%)
Black	23,058 (27.4%)	8580 (25.9%)	2424 (20.2%)	8528 (33.1%)
Hispanic	5442 (6.5%)	2114 (6.4%)	403 (3.4%)	2148 (5.8%)
Asian	5333 (6.3%)	2808 (8.5%)	410 (3.4%)	1478 (5.7%)
Other	5597 (6.6%)	1819 (5.4%)	680 (5.7%)	2139 (8.3%)
Clinical measures				
A1c, mean	57 mmol/mol (14) 7.4% (1.3)	51 mmol/mol (7) 6.8% (0.6)	45 mmol/mol (7) 6.3% (0.6)	74 mmol/mol (14) 8.9 (1.3)
A1c SD, mean	8 mmol/mol (7) 0.7% (0.6)	5 mmol/mol (4) 0.5% (0.4)	4 mmol/mol (4) 0.4% (0.4)	12 mmol/mol (8) 1.1% (0.7)
DCSI, Mean	2.0 (2.06)	1.4 (1.67)	3.8 (2.25)	1.56 (1.72)
BMI (kg/m [2])	33.4 (7.57)	33.1 (7.42)	31.7 (7.34)	34.2 (7.6)
Medications				
Sulfonylurea	40,290 (47.8%)	10,898 (32.9%)	3907 (32.6%)	18,044 (69.9%)
Metformin	56,950 (67.6%)	21,413 (64.6%)	4643 (38.7%)	21,259 (82.4%)
Insulin	22,124 (26.3%)	4410 (13.3%)	2516 (21.0%)	10,977 (42.5%)
Thiazolidinedione	8276 (9.8%)	2023 (6.1%)	542 (4.5%)	4290 (16.6%)
Alpha‐glucosidase inhibitors	729 (0.9%)	135 (0.4%)	53 (0.4%)	384 (1.5%)
Other	1418 (1.7%)	342 (1.0%)	106 (0.9%)	734 (2.8%)
Medication Adherence, Proportion of Days Covered ≥ 80%	58,635 (69.6%)	21,167 (63.9%)	6002 (50.1%)	21,227 (82.4%)
Comorbidities				
Tobacco use	19,591 (23.3%)	6880 (20.8%)	3838 (32.1%)	5409 (21.0%)
Cardiovascular disease	23,946 (28.4%)	7138 (21.5%)	7655 (63.9%)	4299 (16.7%)
Cerebrovascular disease	7536 (9.0%)	1943 (5.9%)	2657 (22.2%)	1302 (5.0%)
Congestive Heart Failure	8878 (10.5%)	1837 (5.5%)	3690 (30.8%)	1363 (5.3%)
Hypertension	67,246 (79.8%)	26,025 (78.5%)	10,772 (90.0%)	19,378 (75.1%)

Abbreviations: A1c, haemoglobin A1c; DCSI, Diabetes Complications Severity Index.

**TABLE A14:** Adjusted hazard ratios (95% CI) predicting mortality by ≥ 60% A1c time in range and ≥ 60% A1c time below range stratified by A1c target ranges.

Variables	A1c target range 42–53 mmol/mol (6.0%–7.0%) (*n* = 53,780)	A1c target range 53–64 mmol/mol (7.0%–8.0%) (*n* = 15,366)	A1c target range 58–69 mmol/mol (7.5%–8.5%) (*n* = 10,947)	A1c target range 64–75 mmol/mol (8.0%–9.0%) (*n* = 4136)
≥ 60% A1c time in range (*n* = 33,137)	1.0	1.0	1.0	1.0
Number of deaths	1827 (3.4%)	1350 (8.8%)	1043 (9.5%)	106 (2.6%)
≥ 60% A1c time below range (*n* = 11,975)	1.03 (0.87, 1.22)	1.28 (1.18, 1.39)	1.08 (0.99, 1.18)	1.66 (1.28, 2.15)
Number of deaths	155 (0.3%)	1979 (12.8%)	2072 (18.9%)	758 (18.3%)

*Note:* Models included all covariates.

Abbreviation: A1c, haemoglobin A1c.
